# Exploring Fire Management Strategies at the Wildland-Urban Interface with an Agent-Based Model Framework

**DOI:** 10.1007/s00267-026-02561-7

**Published:** 2026-08-27

**Authors:** Sven Christ, Richard Sliuzas, Nina Schwarz

**Affiliations:** PLAN Department, ITC Faculty, University of Twente, Enschede, The Netherlands

**Keywords:** ABM, Fire management, Biodiversity, Informal settlement, South Africa, Fynbos

## Abstract

Rapid urbanisation often leads to the growth of informal settlements in hazard-exposed areas. Wildfires are one such hazard that may threaten settlements but, at the same time, are essential for the survival of fire-adapted and fire-dependent wildland species. Fire management needs to navigate the needs of residents and biodiversity conservation within a coupled social-ecological system such as the wildland-urban interface (WUI). In our study, we develop an agent-based modelling framework to explore the potential outcomes of different fire management strategies in a WUI. Residents in the model respond to fires by relocating within the settlement or leaving. Our model simulates Imizamo Yethu, an informal settlement in the City of Cape Town, South Africa, where fynbos is a native and fire-dependent wildland species. Our framework includes feedback between urban growth, fuel load and wildland health, allowing us to investigate trade-offs for different management strategies. Model output indicated that some vegetation types may be surprisingly resilient, also under fire suppression, highlighting areas where future work may be beneficial. The framework can serve as a starting point to investigate other case studies, also with different sub-models for fire spread, vegetation or residents’ behaviour included.

## Introduction

Urban/settlement fires are a growing concern in informal settlements (Karmakar et al., 2024). Current trends of rapid urbanisation in the Global South drive the exposure of many new urban residents to wildfire hazards as well, as they often are forced to choose marginalised informal settlements (Arku and Marais, 2021; Bauer and Scholz, 2010; Christ et al., 2022b). These informal settlements are unplanned and frequently built on hazardous land not intended for urban development (Kahanji et al., 2019; Walls et al., 2019).

Wildfire threatens both ecological systems and the adjacent settlements, yet some wildland species depend on periodic burning (Bond, 1980; Christ et al., 2022b; Cochrane and Barber, 2009; Keeley et al., 2012). Fire-adapted or fire-dependent ecosystems require fire at certain periods to remain ecologically viable (ibid). Fires at these wildland urban interfaces (WUIs), thus, require careful understanding and management to maintain healthy ecosystems while ensuring safety for urban populations (Walls et al., 2017).

**Fig. 1:**
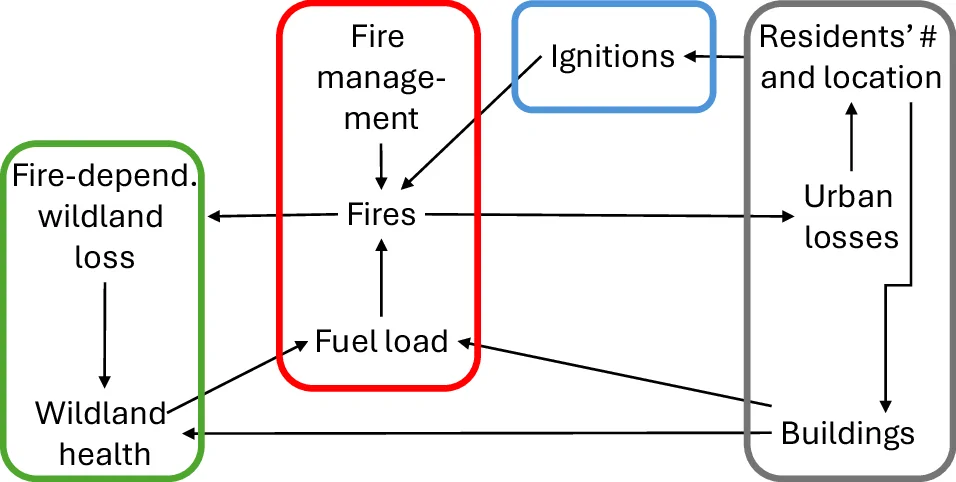
Fire-dependent WUI as a social-ecological system modified after Christ et al. (2022b). Green represents the wildland system, red the fire management system, blue ignitions and grey the urban system

The WUI is an area where the fire outcomes for both wildland and urban areas are tightly linked and create a close feedback loop in a socio-ecological system impacting both residents’ safety and wildland vegetation health (Christ et al., 2022b) (Fig. 1). Figure 1 shows how fire management, such as controlled burning or fire suppression, is a system that has a measurable impact on wildfires that further impacts human settlement systems and wildland systems, which again impacts the choice of fire management.

Various fire management strategies, such as controlled burning or selective firefighting, have been proposed for WUIs with fire-dependent wildlands (Forsyth et al., 2019; Forsyth and Van Wilgen, 2008; Le Maitre et al., 2014). Maintaining a specific fire return interval through controlled burning is often suggested, as it ensures vegetation viability and reduces fuel loads (Van Wilgen et al., 2012). However, some WUI residents oppose controlled burning for fear of fire and smoke covering their homes (Christ et al., 2023; Ferster et al., 2013). Consequently, in some contexts, precedence for protecting urban settlements, both formal and informal, is given, and the strategy adopted by local governments is normally near-total fire suppression (Christ et al., 2022b; Van Wilgen et al., 2012). This strategy endangers residents as over-suppression of fires leads to high fuel loads sustaining large wildfires that cannot be suppressed easily (Frost et al., 2018; Gorte, 2013; Westerling et al., 2006) and is most controversial globally (Christ et al., 2022b; Sayagues, 2015; Van Wilgen et al., 2012). It further endangers fire-dependent wildlands where species are lost if the suppression is longer than the species’ viable age (Ibid). Finally, a compromise of these approaches is using controlled burning of wildlands when appropriate and letting wildland fires run their course when safe to do so (Van Wilgen et al., 2012). More research is still required into management strategies to understand the potential impacts and trade-offs for wildland and residents. The WUI as a socio-ecological system is complex and, therefore, difficult to understand, due to a multitude of interactions at various time scales (Christ et al., 2022b; Schulze et al., 2017).

It has been shown that socio-ecological models can effectively be modelled using agent-based models (ABMs) (Thober et al., 2018), as they enable modelling individual decision-making and can run at varying spatial and temporal scales (Hjorth et al., 2020; Schulze et al., 2017). Several ABMs on different aspects of wildfire modelling have taken up this challenge (Table 1). Millington et al. (2008) investigated commercial and traditional farming practices in a wildfire context. Wildfire as a socio-ecological system in dryland conditions was addressed by Fischer et al. (2013). Fisher et al. (2021) mainly addressed fire spread, fuel, hazard exposure and communication to residents without delving into the broader implications for wildland or the decision-making processes of residents. The work of Spies et al. (2017) mainly focussed on an ABM framework to address the decision-making of decision-makers in USA-based fire-prone landscapes. Further, wildfire-based ABMs include the model by Grajdura et al. (2020), which simulates single fire event-based evacuation scenarios during wildfires, focusing on human behaviour in response to immediate fire threats. While this model provides valuable insights into evacuation dynamics, it does not integrate the ecological impacts of fire management strategies or the long-term wildland health. The hybrid socio-ecological system-based Envision model provides parameters for predicting homeowner responses to wildfire risk but lacks further exploration of how these responses influence ecological outcomes in the WUI (Olsen et al., 2017). Envision was also used as the base for an investigation of land use decision-making and wildfire risk mitigation (Johnson et al., 2023). Further, Ribeiro and colleagues (2023) used a hybrid cellular-automata ABM approach to investigate the impact of fire management on land use change parcels.

**Table 1:** Existing ABMs investigating wildfire

Source	(Millington et al., 2008)	(Fischer et al., 2013)	(Olsen et al., 2017)	(Spies et al., 2017)	(Grajdura et al., 2020)	(Fisher et al., 2021)	(Johnson et al., 2023)	(Ribeiro et al., 2023)
Contextual setting	Agricultural land use comparing commercial vs. traditional farming practices.	Wildfire social-ecological system analysis in a dry forest landscape.	Risk mitigation behaviour among homeowners in the WUI.	Forest management in a fire-prone landscape with a focus on multiple owner groups’ decision-making (federal, tribal, private, etc.).	Wildfire evacuation scenarios	Wildfire simulation model to inform local communities of various fire management strategies and their effects on fire spread patterns.	Wildfire risk and landowner decision-making of land use for fire mitigation	Policy options for wildfire mitigation based on land use change
Geographical region	Agricultural land use in Mediterranean region.	Dry forest landscape in the southwestern USA.	Central Oregon (USA).	Oregon (USA).	California (USA).	Australia, fire-prone.	Rural value floor and foothills Oregon (USA).	Fire-prone regions in Portugal
Scale	Landscape-scale.	Multi-county scale.	Community- and parcel-scale.	Large regional scale.	Resident scale evacuation	Landscape-scale.	Landowner scale.	Stylized land use parcels
Agents	Commercial and traditional farmers.	Land managers, organisations.	Homeowners.	US Forest Service, Bureau of Land Management, corporate forest owners, Tribes, State of Oregon, family forest owners, and homeowners.	Evacuees, emergency managers	Ignitions, fire agents (representing fire spread and behaviour under different management scenarios).	Landowners, vegetation	Land use cell
Drivers of behaviour	Profitability, with differences in land-use decision-making among farmer types.	Scarcity of landscape values and social network dynamics.	Protection Motivation Theory.	Wildfire risk reduction objectives and area targets. Family owners and homeowners, risk reduction combined with personal utility maximization.	Evacuation survey for evacuee behaviour and road network available.	No behaviour modelled.	Climate change, landowner decision-making based on a survey.	No behaviour modelled.

Many of these models have focussed on fire spread, evacuation and resulting hazard exposure, landcover use decision-making and even fire management strategies at various scales. Furthermore, these ABMs have so far been developed for regions in industrialised countries with manifold resources available for fire management. However, ABMs are yet to be used to investigate the potential effects of different fire management strategies on wildland health and individual residents’ location choice decision-making in informal settlements in the Global South. Such research requires an integrated approach that explicitly covers the feedback loops between residents, fires and wildlands at the WUI.

To investigate the impact of two opposing fire and vegetation management strategies - fire suppression and controlled burning and combinations of these - at least three building blocks are required: social (i.e. residents), ecological (i.e. wildland vegetation), and fire. Building on the existing studies, our goal is not to produce a fully validated case-specific model but to propose an adaptable framework linking social, ecological and fire processes and their management at the WUI. The use and value of the framework is demonstrated through a case application in Imizamo Yethu, serving to illustrate potential interactions rather than to forecast precise outcomes. To do this, we compare the potential outcomes of several fire management scenarios with a no-fire-management scenario and a scenario without fire. Our overarching research objective is: To develop an ABM simulation framework that can be used to provide useful insights for policy-making into the interaction of fire management strategies with informal settlement residents’ decisions to stay or relocate and the ecological viability of fire-dependent wildland species.

In previous research, Christ et al. (2023) have shown that the residents of Imizamo Yethu can be characterised with an adaptation of Turner’s (1977) types of residents: dependent bridgeheaders, unattached bridgeheaders, classic bridgeheaders, potential consolidators and classic consolidators. Their research provides a ready-made numerically implementable baseline for the residents’ decision-making. We limit residents’ decisions to deciding to stay in the informal settlement or to relocating within or outside the settlement. A resident’s decision was based on the perceived hazard of wildfire or settlement fire. Perceptions of the impact/hazard of fire on residents have been theorized to influence their decision to take action related to fire events (Christ et al., 2023; Dupey and Smith, 2019). As this study demonstrates a framework and not an in-depth study of one or more actual fire events nor a study of detailed vegetation succession, we have chosen generic parameters for fire based on Fisher et al. (2021) and Jayiya et al. (2004), Le Maitre (2015) and Zahn and Neethling (1921) for vegetation. Using Imizamo Yethu as a case study, we focus on developing a framework to investigate three research questions:

1. What is the impact of fire management strategies on fuel load, fuel continuity and general fire patterns?
2. What is the impact of different fire management strategies on generic vegetation outcomes?
3. What is the impact of fire management strategies on resident decision-making and thereby on their distribution into different Turner types within the settlement?

We emphasise that this study does not aim to develop or validate a physically realistic fire spread model. Instead, fire is represented as an abstract, stylised process to generate spatial dynamics within a coupled social-ecological system. The purpose is to explore interactions between fire, vegetation, and resident decision-making at an aggregate level, rather than to simulate or predict real fire behaviour.

## Study Area

**Fig. 2:**
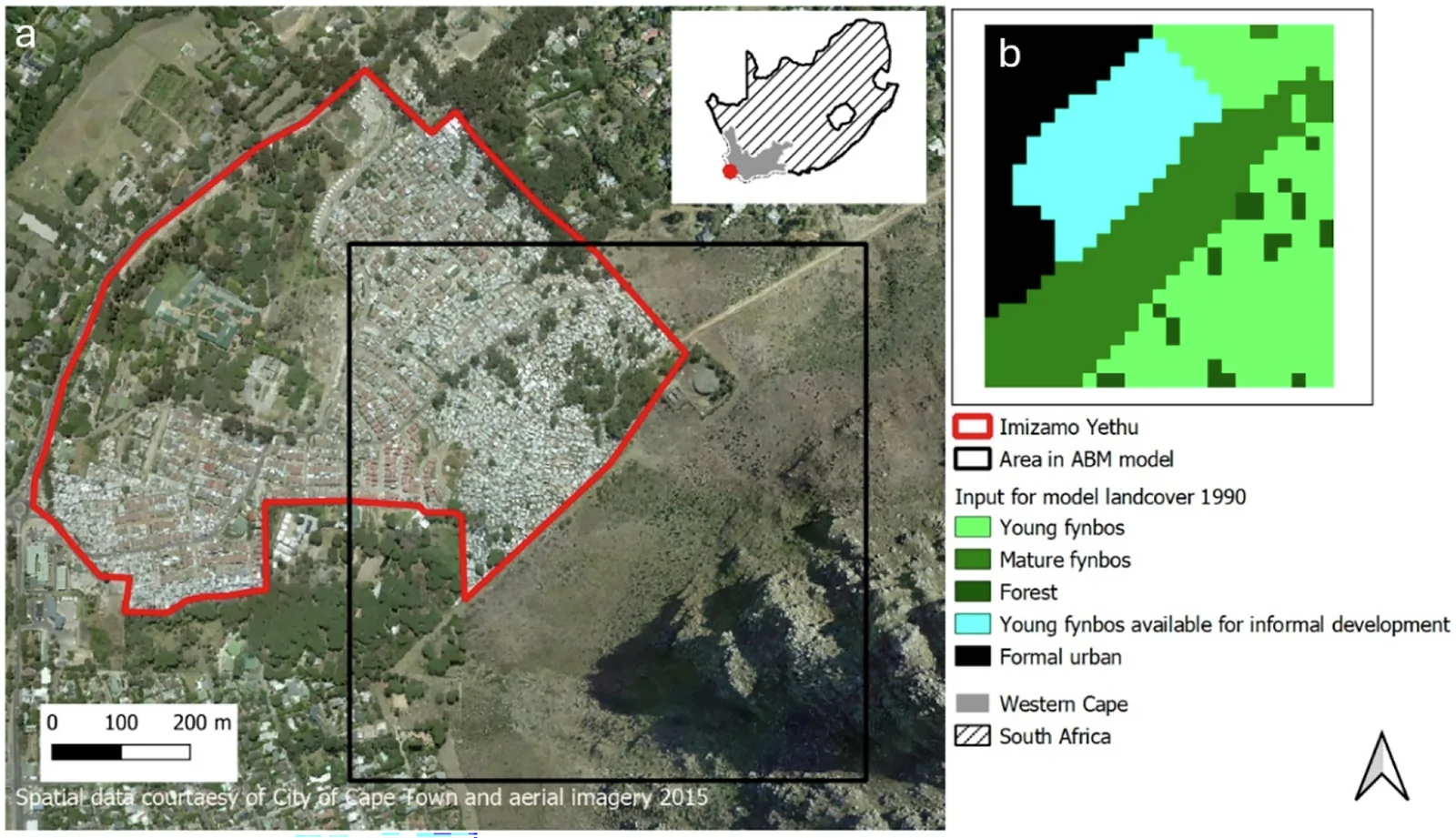
Imizamo Yethu’s location today (**a**) and the modelled inset zooming in on the informal part with 1990 land cover as base for the model (**b**). No grassland visible as this is the landcover in 1990 as per the national landcover 1990 dataset

The City of Cape Town (CoCT) is a Global South city facing fast-growing informal settlements and has one of the highest numbers of threatened wildland vegetation species globally (Christ et al., 2022b; Cinnamon and Noth, 2023; Holmes et al., 2012). The dominant vegetation is the fire-prone and fire-dependent fynbos (Bond, 1980; Van Wilgen, 2013a). Management of fire at the WUI here is about reducing both urban and ecological risks. Imizamo Yethu in CoCT straddles this boundary and is situated at the border of Hout Bay and Table Mountain National Park (Fig. 2). Globally, there is no single definition of the WUI, but in South Africa, 100 m in both directions from the contact zone is often used (Forsyth and Le Maitre, 2015; FynbosFire, 2016; Radeloff et al., 2018; Stewart et al., 2007).

The national park administration supports CoCT’s Integrated Development Plan by preserving the natural environment, offering recreational opportunities, and enhancing residents’ quality of life, including ensuring safety, in alignment with the National Environmental Management: Protected Areas Act (Table Mountain National Park management et al., 2015). Large areas of the national park still either burn too often or not frequently enough (Christ et al., 2022b; Forsyth and Van Wilgen, 2008). This is also reflected in the Table Mountain National Park Management document indicating there are too frequent fires from negligence and malicious intent and too infrequent fires due to local and municipal restrictions.

Imizamo Yethu (IY) was established in the early 1990s by black workers in the Hout Bay area towards the end of the oppressive Apartheid regime (Christ et al., 2023). The settlement has experienced multiple settlement fires and escaped fire from the settlement into the wildland over the years; however, it remains a viable location for many residents (Ibid). Wildfires have also threatened the settlement, such as in February 2006 and again in 2015 (African News Agency, 2015; Harte et al., 2009). In 2022, a settlement fire also escaped IY into Table Mountain National Park and was fortunately quickly suppressed (Cape Etc, 2022). This combination of a vulnerable informal settlement and a national park with fire-dependent vegetation has been selected as the study area for this research to investigate the impact of varying fire management strategies. Although some argue that these residents have no choice but to remain in the same informal settlement after a fire event, research has shown that even under perceived and realised constraints, the decision to stay in a location is made with a sense of agency (Hofstede et al., 2023). Furthermore, many South African informal settlement residents moved from the former black homelands, which often lacked economic opportunities (von Fintel and Fourie, 2019). While perhaps not ideal locations either, they do represent a location to return to if the perceived hazard of the urban informal settlement became larger than the perceived rewards of staying.

## Methods

This study was conducted using an ABM-based framework, with its concept and implementation detailed in section ‘Framework and implementation’. Section ‘Parameterisation’ describes the parameterisation, section ‘Validation’ the validation, and section ‘Management strategies’ lists the management strategies and their implementation details. A full ODD+D (Mueller et al., 2013) documentation of the model is available on Comses (https://www.comses.net/codebase-release/33269c5b-6edb-41e4-8330-e0dd67ed85b0/).

### Framework and Implementation

Based on Fig. 1, we propose the following modules of a simulation model to investigate the interaction of fire management strategies with informal settlement residents’ decisions to stay or relocate and the ecological viability of fire dependent wildland species: (1) The resident module simulates residents’ decision making regarding where to settle and whether to respond to fires by leaving or relocating within the settlement (section ‘Residents’). (2) The vegetation module simulates vegetation dynamics over time, responding also to fires and fire management strategies (section ‘Vegetation dynamics’). (3) The ignitions module simulates ignitions in the study area (section ‘Fire ignitions’). (4) The fire spread module simulates the spread of fires and how management influences them (section ‘Fire spread and fire management’). (5) The controller module steers the simulation by controlling the flow of information between modules and triggering modules (section ‘Controller module and simulation flow’). This approach aligns with the work of Sun et al. (2016) that suggests a pattern-oriented modelling approach that is modular for complex and complicated modelling scenarios.

In the following sections, the modules are explained in more detail. Our approach for designing or choosing the modules was to focus on the characteristics of fire spread patterns and their dynamics with vegetation and residents’ decision-making. In principle, all of the modules can be individually replaced for more detail or different hazards; human decision-making can be investigated and parameters calculated based on either empirical fieldwork or generic decision-making as long as these can be converted into mathematical formulae. The intent of this study is to highlight the interactions and feedback among management strategies, fire, vegetation and residents’ decisions as a proof of concept. These areas are often treated in isolation within their own disciplines.

The fire module represents the characteristic spread patterns that emerge under varying fuel loads, management strategies, and settlement configurations, rather than specific fire behaviour. The focus here is on the spread of large fires, not small fires only affecting one or two individual homes. This fire module should only be used as an example of how fire patterns interact with the other modules. Any future work focussing on realism or policy implementation should replace this with a fire module or modules appropriate for their study area, in terms of vegetation and formal or informal homes.

**Fig. 3:**
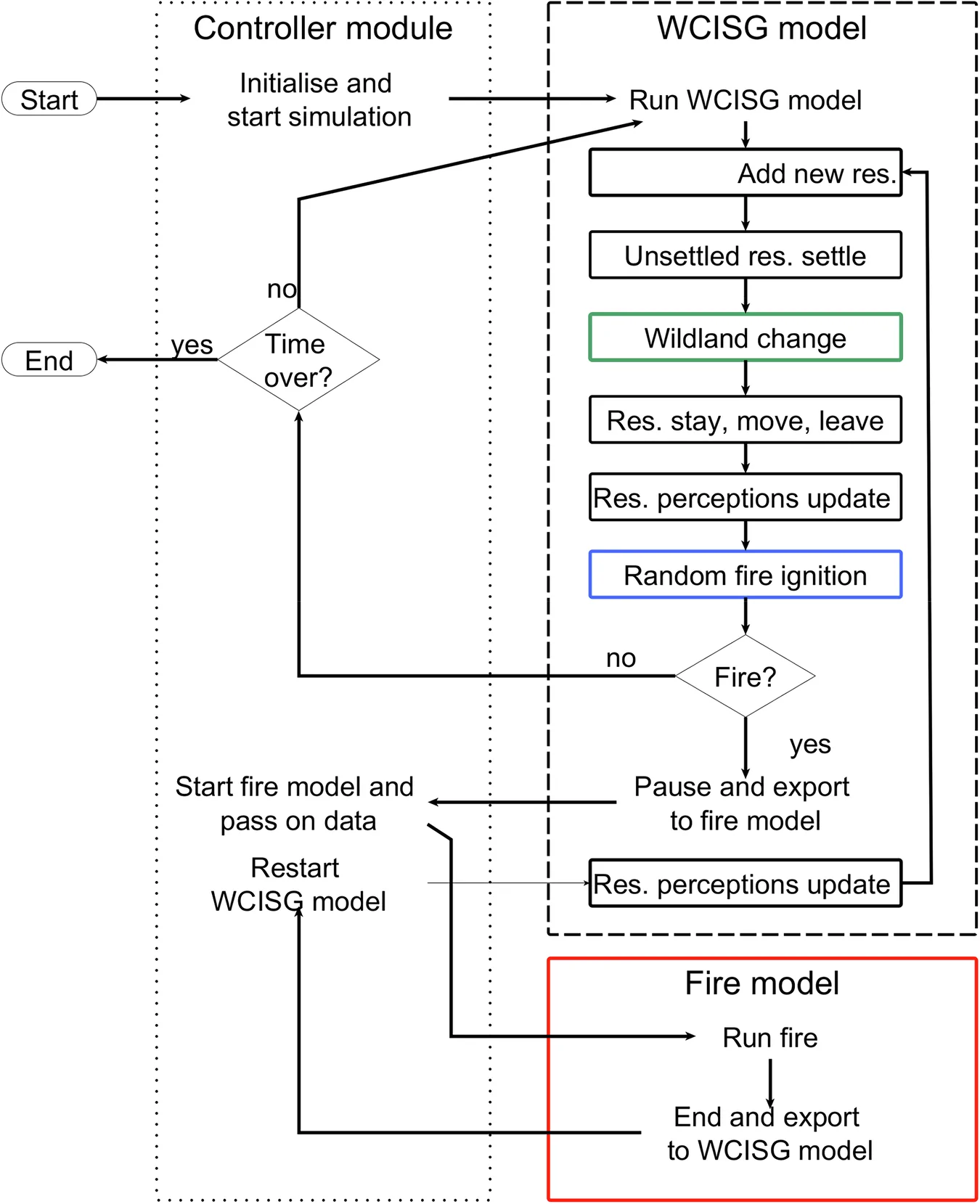
Fire-WCISG overview with controller (dotted line), WCISG model (dashed line) and the fire model (red). WCISG processes for residents are dark grey, for wildland green and for ignitions blue. Res.: Residents

We implemented the framework as the Fire-Wildland Change and Informal Settlement Growth (Fire-WCISG) model in NetLogo version 6.0.4 (Wilensky, 1999). As wildland change and residential decisions occur over months and fire events over hours, the LevelSpace extension was used to couple models (Hjorth et al., 2020). The Fire-WCISG model simulates the interactions between residents of the informal settlement, the surrounding wildland and fires igniting and spreading in both the urban and wildland (Fig. 3). Fire-WCISG is composed of three parts: (1) a controller module, (2) a newly developed urban model named Wildland Change and Informal settlement growth (WCISG) that encompasses the modules of residents, vegetation and ignitions and (3) the Fisher et al. (2021) fire model that simulates rough approximations of fire spread.

#### Controller Module and Simulation Flow

The controller module is the model interface used for the WCISG and fire models to run separately and interact. The controller initiates the WCISG model and lets it run until it detects a fire. The WCISG model is responsible for residents entering the model, sensing and interacting with wildland. Furthermore, WCISG implements wildland change and ignites fires. Once a fire is detected by the controller model, it pauses the WCISG model and initialises the fire model, which runs until the fire is exhausted. At this point, the controller model passes on the burned area dataset to the WCISG model and continues running it either till the next fire is detected or the end of the model run time. For full details on individual model processes and decision making, please see the extended ODD+D documentation at Comses.

The fire module operates at a different temporal scale than the others: One time step in the WCISG modules represents 3 months and encompasses residents’ locational choices, fire ignitions, and the vegetation in the wildland maturing. In the fire module, one time step represents 3 h as it was developed (Fisher et al., 2021).

#### Residents

The framework requires residents whose movement changes settlement extent and density and whose decisions are influenced by fires occurring. To conceptualise and parameterise that module, we conducted empirical surveys documented elsewhere (Christ et al., 2023, 2022a) and focus here on the model concept. Empirical data on other contexts could be used to inform alternative models on residents.

In the WCISG model, residents are agents moving into the settlement, moving within and leaving the settlement. Residents belong to one of five types as identified by Christ et al. (2023) based on Turner (1977) for the IY settlement: classic bridgeheader, dependent bridgeheader, unattached bridgeheader, classic consolidator and potential consolidator, each having their own characteristics. Classic bridgeheaders are very low-income residents brought into an area for the economic benefits it offers. Dependent bridgeheaders are typically unemployed and, while they may seek the economic benefit of the area, they are dependent on the support of others. Unattached bridgeheaders are low-income residents, but have employment not linked to the area. Classic consolidators are established residents with stable income and the ability to start securing their lifestyle, while potential consolidators are gaining employment stability but not yet focussing on stabilising their lifestyle (Christ et al., 2023).

Residents decide where to move within the settlement and leave the settlement based on Turner’s theory (1977), place attachment (Ghasemi et al., 2020) and Protection Motivation Theory (PMT) (Brenkert-Smith et al., 2012; Dupey and Smith, 2019; Martin et al., 2007; Rogers, 1975). Influence factors based on these theories were determined for the study area in Christ et al. (2023). Five multi-attribute functions (see section ‘Parameterisation of residents’ decision-making’) calculate the probability for each agent to stay, leave or move, and the option with the highest probability is chosen. After a fire, residents are informed if their homes were burned or that there was a fire in the settlement. This information updates residents’ risk perception based on the PMT and place attachment variables and their change after fire as described in Christ et al. (2022a, 2023). The parameters that are adjusted are coping self-efficacy, coping response-efficacy, coping cost, threat appraisal individual, threat appraisal community, threat appraisal direct, place attachment and threat severity. The adjustment depends on whether a resident lost their home in the fire or not.

Residents moving to or within IY can select either an unoccupied house or build a new house on available land outside of Table Mountain National Park. Plots are available as long as the density limit of 62 per pixel (30 × 30 m) is not reached; this was based on the highest density reported in an informal settlement in Mumbai by Das et al. (2021) of about 68,900 persons/km². Further details on residents’ movements are given in ODD+D protocol section ‘Study area’.

#### Vegetation Dynamics

The WCISG model uses a simplified vegetation dynamic in the wildland. Our key consideration was to mimic the overall process of how fire-dependent wildland interacts with fires and urban growth. Therefore, vegetation is treated at the level of broad fynbos functional types, parameterised using common aggregate values. The age for fynbos maturity (7 years) in our study was taken from the work of Kraaij et al. (2013) and Kraaij and Van Wilgen (2014), which indicated that while there is large variability between the maturation of species both in terms of location and type, fire return over 7 years always left more than replacement levels of fynbos. Seven years also represents the age where fynbos is assumed to be able to sustain a fire. We recognise that our modelled approach does not explicitly distinguish between fynbos species, including different types of reseeders and resprouters, nor does it incorporate detailed rainfall or climate variability’s impact on fynbos. Furthermore, no invasive species are considered in the model, although some invasive species also benefit from fires. Grassland in our study is treated as a mixed vegetation state that can become fynbos or forest depending on the dynamics described below. Forest is treated as a generic vegetation state of older vegetation from which fynbos can never return.

In the WCISG model, the vegetation dynamics are simplified as follows: After a fire, grassland will establish with an initial fuel load of 0. If fynbos seeds are present, grassland transitions to young fynbos after 4 years, which becomes mature fynbos at 7 years and begins seeding (Van Wilgen et al., 1992). Mature fynbos grows until 21 years, after which the fynbos seed dies and, in the modelled landscape, becomes forest as the next broad category of vegetation. This was chosen as it has been shown that optimal fire regimes are often around 10–20 years, with there being a decline around 12 years and total senescence occurring around 30 years (Van Wilgen et al., 1992). If fynbos seed burns twice before becoming young fynbos, the seed will be lost. Without fynbos seeds present, grassland will turn into forest after 21 years without fire. Forest is the final vegetative stage and continues to grow in fuel load. These are all generalist interpretations and not species- or location-specific interpretations. To have a detailed model of vegetation succession from burned area to mixed invasive and individual fynbos species to true forest would take more complex calculations than is possible in this proof-of-concept framework.

Wildland fuel growth was developed as a regression model (Fuel = −0.035 + (0.081 * vegetation age)) derived from the fuel load growth data found in mixed fynbos vegetation (Jayiya et al., 2004; Le Maitre, 2015; Zahn and Neethling, 1921); one value was used regardless of vegetation type in the vegetation cover, as fuel is modelled as a function of vegetation age. This also partly accounts for the lack of climate data that influences fuel load growth overtime. Fuel load for the informal area was calibrated to an equation using the number of homes in the location (see Table 2 for all settings). Further details on vegetation dynamics are given in ODD+D protocol section III.iii.

**Table 2:** Parameterisation of wildland and fire parameters for baseline model, with urban and wildland suppression as the only management strategies

Model parameter	Explanation	Setting	Reference / data analysis
Percentage_succesful_ wildlandfire_suppression	How effective the suppression should be of an ignition that occurs in the wildland. If suppression is not successful, the ignition is deemed a fire and passed on to the fire model.	80%	Calibrated to Christ et al. (2022a, 2022b).
Percentage_succesful_ urbanfire_suppression	How effective the suppression should be of an ignition that occurs in the urban settlement. If suppression is not successful, the ignition is deemed a fire and passed on to the fire model.	90%	Calibrated to Christ et al. (2022a, 2022b).
Number_urban_ignitions_ per_tick_per_hectare	The probability that an ignition occurs in the urban area per time step based on the size of the urban area.	0.25	Based on (GeoTerraImage, 2016)
Number_wildland_ignitions_ per_tick_per_hectare	The probability that an ignition occurs in the wildland area per time step based on the size of the wildland area.	0.03	Based on (GeoTerraImage, 2016)

#### Fire Ignitions

Finally, the WCISG model simplifies fire ignitions as random processes. In the informal urban area, ignition likelihood increases with settlement density, as higher density implies more flammable material but also more hazardous sources of energy and light, including candles, paraffin stoves and open fires (Pharoah et al., 2022; Wang et al., 2021). The wildland uses a separate formula that was based on the number of fires per hectare as calculated for CoCT reported fire incidences. Both wildfire and informal settlement fire statistics are derived from fire data in the CoCT fire incidence dataset (up to 2021), respectively, available via the OpenData portal (https://odp-cctegis.opendata.arcgis.com). Fire ignition does not guarantee fire spread, as the model has a statistically modelled suppression mechanism based on the probability of historically successfully suppressed fires (see ODD+D section B.III.iv). If suppression fails, the fire module is triggered.

#### Fire Spread and Fire Management

Fire management is selected and simulated within the WCISG model, with suppression of initialised fires and controlled burning also handled in the WCISG model. If an ignition was not statistically suppressed, it is handed to the fire module. Ignitions once passed to the fire module are allowed to run until they naturally terminate.

Models simulating fire spread are available for different contexts and purposes (Ghodrat et al., 2022; Singh et al., 2024). Physics-based models such as FARSITE (Finney, 1998) include detailed processes, e.g. fuel load, fuel moisture and meteorological conditions. More detailed models can represent individual building structures or even leaf structure (Krix and Murray, 2022; Waly et al., 2026). Accordingly, the spatial and temporal resolution of these models varies by the level of detail required. This can range from sub-building to large parcel and from seconds to hours. While these models are able to approximate large-scale fire spread patterns with reliable accuracy, they are data intense whereas simpler proxy models such as Fisher et al. (2021) are based solely on the spatial distribution of fuel load and coarse meteorological conditions. For instance, the Fisher et al. (2021) model was designed to simulate fire spread in Australian landscapes at a spatial resolution of 30 × 30 m and a temporal resolution of about 3 h, with limited data requirements. For this work, we chose the Fisher et al. (2021) fire model for demonstrating our own framework for the following reasons: First, it is compatible with the abstract vegetation representation used in this framework. Second, a simple fire model allowed us to focus on the system-level interactions without additional uncertainties introduced by processes such as structure-to-structure fire or detailed meteorological interaction. This choice made the sensitivity analysis more manageable. Third, some studies have suggested similarities between large-scale fire spread patterns of burning wildland and burning informal settlements (Stevens et al., 2020; Walls et al., 2017). These similarities refer to broad patterns, not fire spread between a small number of informal homes. Due to the rough approximation, the model employed here should by no means be used to predict fire spread in an actual firefighting situation or direct policy action, as the model is built on hypotheses. Any future work based on our framework must replace the fire model with the appropriate model for their study area and design.

Like the original model, our adaptation of Fisher et al. (2021) runs on a 30 × 30 m grid with grid cells characterised by landcover and the fuel thereof. This links with the vegetation grid cell that the entire model runs on. Fuel for wildland is determined by vegetation age, and for the settlement it is determined by the number of homes on the cell. As a single cell can contain more than one home, the fire module does not model single structure fires or fires affecting only a few homes. These are considered part of the early suppressed ignitions and not modelled. This means only larger fires are simulated here, in line with the aim of the study to investigate the interactions between residents, wildland and fires.

Due to the use of LevelSpace and the controller model enabling communication with the WCISG 3-month temporal resolution, minimal modification was required. The limited adaptations from the original model to our case study are importing the landcover map and the ignition locations that are modelled in the WCISG model. The fire module is initialised by the controller module that monitors the fire runtime to stop when the fire is exhausted. Then, it returns the burned area to the WCISG model.

Our model framework allows for exploring variations and combinations of two distinct fire management strategies: suppression and controlled burning (section ‘Management strategies’). In line with our generic framework approach, controlled burning is modelled idealistically with vegetation that reaches the selected controlled burning age treated as burned without running a fire, limiting the model’s ability to model runaway controlled burning events. This decision was taken as the model does not include active firefighters. Further details on the implementation of fire spread and fire management are given in ODD+D protocol sections B.III.iv.

#### Spatial and Temporal Scales

In this study, a model run lasts for 31 years (1990 to 2021) to be in line with existing described land cover and fire data (Christ et al., 2022b). The landscape (covering an area of 0.585 km^2^) is represented by a grid of 26 by 25 cells, each covering an area of 30 × 30 m (900 m^2^ total area), and distinguishes the following generic land covers: grassland, young fynbos, mature fynbos, old fynbos, urban informal and urban formal (Fig. 2b). It must be noted that it is conceivable that fires or processes outside the study area such as wildfires outside the study area may affect modelling in the real world. Our study here was focussed more on modelling and investigating the patterns of interaction than realism. The 30 × 30 m spatial resolution represents an abstraction of aggregated settlement and vegetation conditions and is not intended to model individual shelter-to-shelter fire dynamics.

### Parameterisation

For this study, the initial landscape, residents and wildfire parameters had to be parameterised. The resident data as well as landscape and fire data were all derived from real-world data as described below and in more detail in the ODD + D documentation.

#### Initial Landscape

The input landcover dataset at initialisation is based on the 30 × 30 m 1990 national landcover dataset of South Africa (GeoTerraImage, 2016). The landscape is divided into areas of formal urban land, areas of young fynbos, mature fynbos, small-scattered forest and fynbos that can be converted into formal land. Forest age is set at 21 years in line with the generic interpretation of our vegetation module; the age for young fynbos is randomly set between 4 and 6 years and mature fynbos between 7 and 20 years based on the interpretation we use for these vegetation categories as described in section ‘Vegetation dynamics’. The vegetation age will dictate the available fuel load and is described in section ‘Parameterisation of wildland and fire dynamics’.

#### Parameterisation of Residents’ Decision-making

Resident decision-making in the model follows the framework for residential choice under fire risk and informal settlement resident types proposed by Christ et al. (, 2023). Residents are parameterised using two surveys: one baseline survey after 9 months without fires in the settlement (Christ et al., 2023), and a smaller survey 1.5 months after a settlement fire (Christ et al., 2022a). The parameters used can be seen in Table 3. The only decision residents make is whether to stay, leave, or move within the settlement based on the perceived fire hazards. The surveys incorporated elements of PMT, place attachment and Turner’s theory for residential choice. Christ et al. (2023) combined the nine remaining variables into multiple linear regression models predicting residents’ decisions (Table 3). Five multi-attribute functions were created, one per decision. Each function sums up nine products, representing concepts such as coping self-efficacy and individual threat appraisal. The regression weights from the empirical survey across all resident types are used as factors of these products (Table 3). Resident attributes are drawn from a database of residents of the five identified Turner types, where the model asks for e.g. a classic bridgeheader; it will randomly select these values from a classic bridgeheader that was surveyed by Christ et al. (2023).

**Table 3:** Regression weights table for decision making, perception change and perception recovery based on the empirical survey and impact of fire on perceptions (Christ et al., 2023)

	Regression weights					Parameter change		Recovery rate post fire
*Decision or action*	*Always*	*wildfire*	*Settlement fire*	*Wildfire*	*Settlement fire*	*Direct fire experience*	*Indirect fire experience*	
Parameter name in model	Stay weight	Move within	Move within	Leave	Leave	Percentage of current value modified	Percentage of current value modified	
Coping_self-efficacy	−0.045	0.223	0.509	0.608	0.415	0.664	0.719	1.103
Coping_response-efficacy	0.169	0.568	0.338	0.428	0.671	0.571	0.634	1.132
Coping_cost	0.214	−0.067	0.039	−0.117	−0.067	1.391	0.860	0.958
Threat_appraisal_individual	−0.392	−0.047	0.037	0.142	0.047	0.865	0.678	1.076
Threat_appraisal_community	0.265	0.213	0.017	0.033	0.139	1.151	0.701	1.025
Threat_appraisal_direct	0.007	−0.008	−0.112	−0.117	−0.122	0.544	0.370	1.138
Place_attachment	0.400	0.105	0.077	−0.083	−0.164	0.997	0.539	1.077
Threat_severity	0.177	0.066	0.071	0.085	0.113	0.881	0.664	1.076
Rewards_of_staying	−0.171	−0.107	−0.048	−0.065	−0.054	No change	No change	No change

To account for changes in residents’ risk perceptions after a fire event, the nine parameters seen in Table 3 are modified by the average percentage impact from the surveyed population before and after the studied fire events, as shown in Christ et al. (2022a). The percentage change of the resident parameters was calculated as the mean difference between the baseline study and those households who lost their homes during the fire and those who did not lose their homes but saw the effects in the community. The percentage change is for all residents based on the individual existing values that they individually hold. The differentiation of impact is based on whether the residents directly lost their home or did not. The variable fire decay is set to 0 and is responsible for the post-fire recovery of perceptions that is limited to four time steps (1 year) to recover the residents to about their pre-fire state. The rate at which recovery is calculated is shown in the recovery rate section of Table 3. This recovery is based on the average impact a fire event has, divided by four time steps, and may be permanently impacted if two fires are experienced before full recovery.

Due to differing settlement growth patterns during the late apartheid era and the post-apartheid free growth period (Christ et al., 2022a, 2022b), no single curve or growth rate could accurately capture the growth rates of both periods. Therefore, two growth rates were calibrated for the residents. The first period is 1990 (initialisation) to 2001 (first census), and then the period thereafter. The calibration was based on the population density in the 2001 and 2011 census for IY. The percentage share of resident types was aligned based on the 2021 survey by Christ et al. (2023). Table 4 shows the number of residents added per time step for population growth of the five resident types (for detailed results see Appendix A.2).

**Table 4:** Number residents added to the simulation per time step per growth period

Resident type	Period 1 1990 to 2001	Period 2 after 2001
Dependent bridgeheader	1	7
Unattached bridgeheader	4	11
Classic bridgeheader	3	13
Potential consolidator	4	37
Classic consolidator	4	16

#### Parameterisation of Wildland and Fire Dynamics

For wildland ignition numbers, we used CoCT fire incidence data between 2009 and 2016, as wildfires (named vegetation fires in the dataset) were not reported for later time periods. For urban ignition numbers, we used CoCT fire incidence data between 2019 and 2021, as the earlier dataset did not contain an estimated damage value for us to extract the number of structures affected per fire incident (GeoTerraImage, 2016; also see ODD+D section B.III.iv for more information). The number of ignitions per year per hectare in CoCT was calculated based on the number of wildland or settlement fires, respectively, occurring in the wildland or settlement. We also used these datasets initially for suppression rate; however, these settings gave unrealistic burned area results. Thus, these parameters were calibrated to the fire patterns in the study area as derived from the fire data in Christ et al. (2022a, 2022b), by running multiple settings until these matched well with burned area size and return intervals as documented in Appendix A.1.

To calibrate the successful fire suppression ratios, we varied Urban Suppression between 85 and 95% in 5% steps and wildland suppression between 50 and 90% in 10% steps per parameter combination. Then, we compared the simulation outcomes with empirical values for average maximum burned pixels, average median burned pixels, average total burned pixels, and a plotted normal distribution. The empirical patterns were determined based on the fire data for CoCT as described by Christ et al. (2022a, 2022b).

### Validation

Four types of validation are distinguished in the literature: input validation, process validation, descriptive output validation and prescriptive output validation (An et al., 2021). Due to the exploratory framework-oriented nature of this study, data availability and time constraints, we focussed on input validation and descriptive output validation. Input validation checks whether the external input to the model is empirically meaningful and appropriate for the purpose. We have critically discussed the empirical basis for the decision-making model in Christ et al. (2022a, 2023) and have elaborated on the input data in the ODD section II.i. Descriptive output validation checks whether model output aligns with the sample data used to build the model. The key indicators we focussed on were the outcomes of the residents, fire patterns and landscape. Results are provided in Appendix A sections A.1 and A.2. For fire especially, we focussed on general patterns rather than reproducing specific fire events or fine-scale fire behaviour. The modelled fire is an analogy rather than a predictive model. We only focussed on whether the number of pixels burned in the period aligned with the number of pixels burned in the same period in reality over the period aligned with the model outcomes. This approach links model results to coarse-scale outcomes and not specific building-structure-based outcomes.

### Management Strategies

The Fire-WCISG model was used to simulate four different fire management strategies: Urban and Wildland Suppression (the current management strategy in IY), Urban Suppression, Urban Suppression with Controlled Burning and Urban and Wildland Suppression with Controlled Burning. In addition, scenarios without fires (total fire exclusion) and no management at all were simulated (Table 5).

**Table 5:** Fire management strategies in the Fire-WCISG model

Fire management strategy	What changes
Urban and Wildland Suppression	Urban Suppression and wildland suppression turned on.
Urban Suppression	Only Urban Suppression turned on. All wildland ignitions allowed to burn.
Urban Suppression with Controlled Burning	Only Urban Suppression turned on. All wildland ignitions allowed to burn. Controlled burning is turned on to burn vegetation at 8 years of age, except forest.
Urban and Wildland Suppression with Controlled Burning	Urban Suppression and wildland suppression turned on. Controlled burning is turned on to burn vegetation at 8 years of age, except forest.
No Fire	Model runs with no fires created.
No Management	All suppression turned off, all ignitions allowed to burn. Controlled burning turned off.

Note: All settings not mentioned here remain as discussed above in the initial landscape and parametrization sections.

To investigate the results and compare the impact of the different strategies on our outcomes, we used ANOVA tests and Tukey’s HSD to find out which results were significantly different (Bogani et al., 2023). To compare the burned area results, we used the Gini coefficient, mean, standard deviation and maximum.

## Results and Discussion

The following sections present and discuss results that could be derived from the framework on the potential impacts of management strategies on fuel load (section ‘Impact of management strategy on fuel load’), fire patterns (section ‘Impact of management strategies on fire patterns’), vegetation outcomes (section ‘Impact of management strategy on vegetation outcomes’) and household number (section ‘Impact of management strategies on household number and type distribution’). Results in these sections illustrate the type of insights that can be generated through the framework rather than definitive forecasts for Imizamo Yethu. We then discuss challenges in implementing a socio-environmental system framework (section ‘Challenges in implementing a socio-environmental system framework’) and derive directions for future research (section ‘Future research’).

### Impact of Management Strategy on Fuel Load

**Fig. 4:**
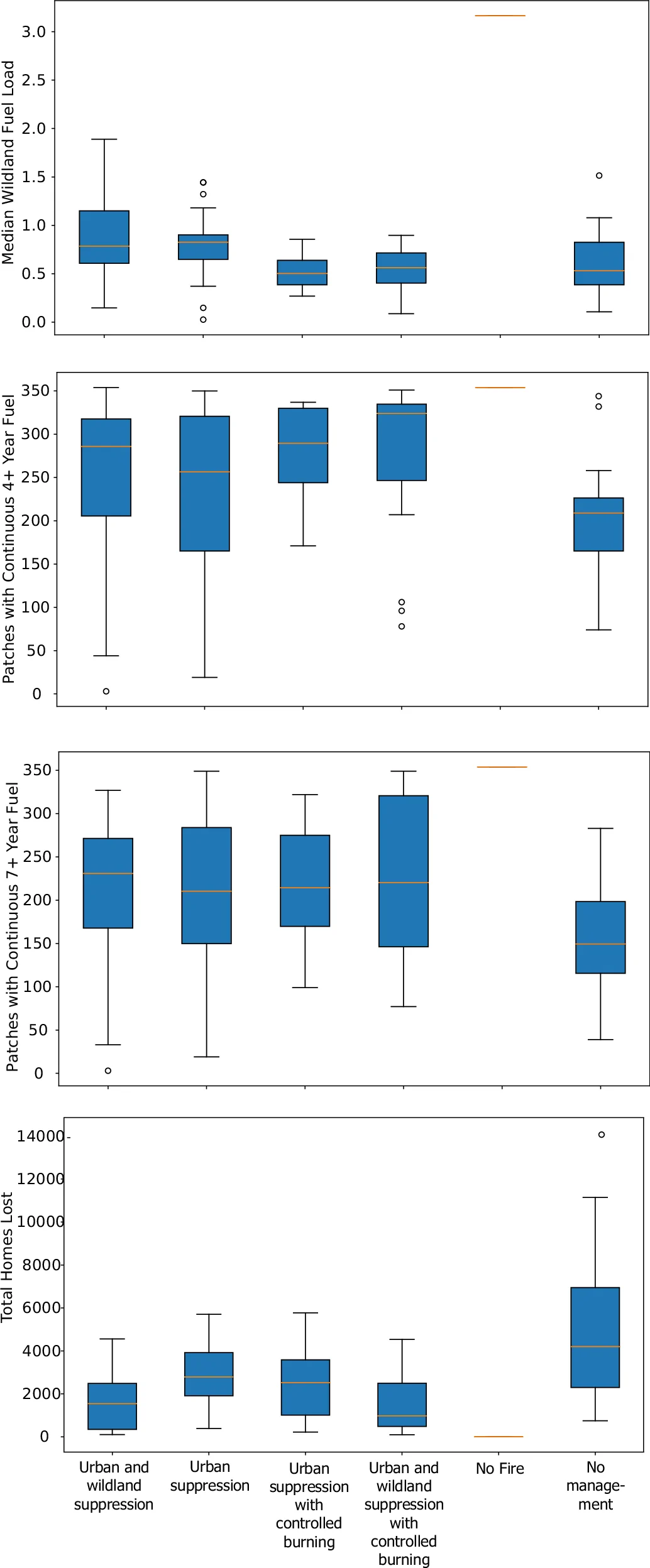
Key model output at the end of 124 ticks (31 years) across 20 runs per strategy: **a** median wildland fuel load, **b** patches with continuous 4+ year fuel, **c** patches with continuous 7+ year fuel, **d** total homes lost

Our model outcomes are consistent with the finding that fuel load increases with time since last fire (Blarquez et al., 2010) as the extreme run with *No Fire* shows the highest level of available wildland fuel (Fig. 4a). Regarding the other strategies, the post hoc Tukey’s test (Appendix A.1) after a significant ANOVA indicates a statistically significant difference between the practice of *Urban and Wildland Suppression* and *Urban Suppression with Controlled Burning* and *Urban and Wildland Suppression with controlled burning*. Except for *No Fire*, none of the other strategies were statistically different to *No Managemen*t, indicating that a lack of suppression may moderate fuel loads as good as any other strategy. In our model, fire keeps wildland fuel down; however, allowing fires to burn may not be the most desirable management strategy close to urban areas. The results suggest that using controlled burning to reduce fuel loads could be a viable strategy. For broad fire management, higher fuel loads have been shown to lead to larger, more intense fires that can be harder to fight (Brando et al., 2016; Cansler et al., 2019). This was evident in the difficult-to-suppress fire on Table Mountain in 2021 (Bega, 2023).

Besides total fuel load, fuel continuity is a secondary factor that influences fire spread: the more continuous fuel loads are, the higher the possibility of spread (Neumann et al., 2022). Evaluating the modelled continuous fuel load over 4 years and over 7 years, however, shows very similar patterns for all management strategies (Fig. 4b, c, and Appendix B.1). The extreme *No Fire* runs are statistically different from all strategies. In line with total fuel load, *No Management* showed also the lowest level of continuous fuel over 4 and over 7 years. This strategy statistically only differs from *Urban and Wildland Suppression with Controlled Burning*. These results point to *Urban and Wildland Suppression with Controlled Burning* having potentially better outcomes in the event of a fire both in terms of fuel load and fuel continuity, under the assumption that *No Management* would be undefendable in a WUI. It is key to mention, though, that while controlled burning-based management strategies also have high fuel continuity, they have a lower overall fuel load and, thus, fires are easier to suppress.

### Impact of Management Strategies on Fire Patterns

It is important to emphasise that the simulated fire outcomes do not represent real fire events but rather stylised fire patterns emerging from simplified assumptions on fuel load and continuity. In our stylised modelled environment, the spatial distribution of fires across the various strategies shows that in the event of an ignition, each informal or wildland cell in IY burns on average once over the course of one simulation (Table 6). *Urban and Wildland Suppression with Controlled burning* has the lowest average of 0.7, while *No Management* has almost 2, and this difference is statistically significant. The average number of burns per pixel of the other strategies falls between these two extremes. This aligns with literature that controlled burning may have better outcomes overall in terms of burned area and fuel load, even though residents are often afraid of it (Christ et al., 2022b; Forsyth and Van Wilgen, 2008; Van Wilgen, 2009).

**Table 6:** Statistics on average number of times a pixel burned per model run

Strategy	Gini coefficient	Mean	Standard deviation	Maximum
Urban and Wildland Suppression	0.16	1.41	0.40	2.7
Urban Suppression	0.15	1.65	0.44	3.25
Urban Suppression with Controlled Burning	0.21	0.88	0.33	2.2
Urban and Wildland Suppression with Controlled Burning	0.24	0.72	0.30	1.6
No Management	0.21	1.88	0.72	3.95

The fire distribution is measured as the count of burns per cell over the simulation period of 31 years, averaged across 20 model runs per management strategy.

**Fig. 5:**
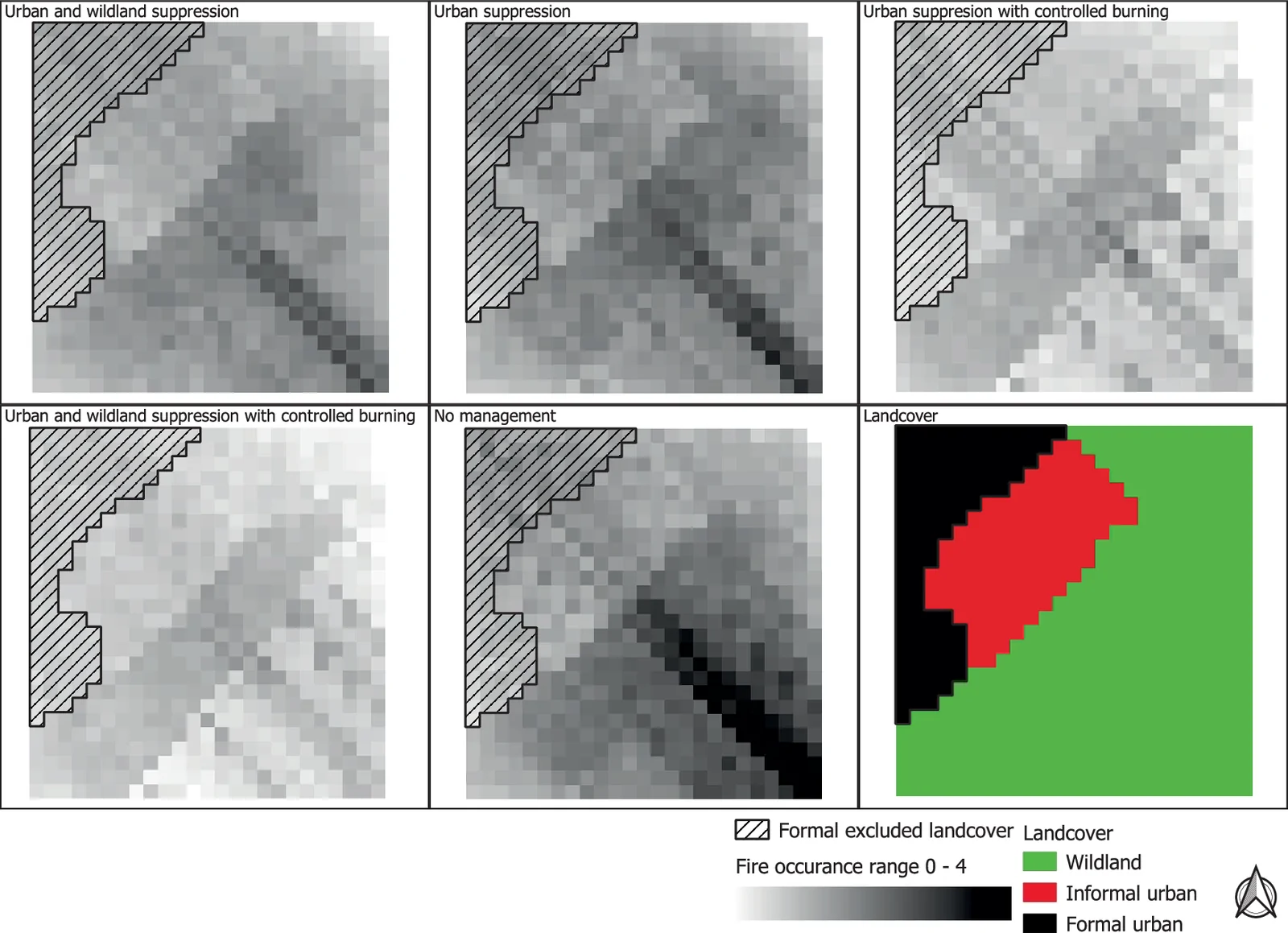
Average fire distribution in the informal and wildland area over 20 model runs per management strategy

The average fire distribution per strategy is visualised in Fig. 5. The burned area patterns for *No |Management* and *Urban Suppression* look very similar, while the two other strategies and their combinations show different patterns. Thus, fire management in the wildland has an impact on urban burned areas. It is also evident that *Urban and Wildland Suppression* still allow burns in the urban area. This model result is in line with empirical evidence (Christ et al., 2022b, 2023) that IY is not fire-free even with the current urban and wildland suppression management style. Furthermore, interesting to note is the band of more burns just outside the informal urban, i.e. the WUI, that is present in all strategies to a greater or lesser extent. This is only absent in the *No Management* strategy. *No Management* has the highest standard deviation of burns per pixel, indicating the highest variability. It is significantly different from *Urban and Wildland Suppression with Controlled burning* showing the lowest variability; the other strategies fall between the two. The other two indicators to describe fire patterns, i.e. the Gini coefficient as a measure of dispersion and the maximum of burns, show differences but none are statistically significant.

While few statistically significant differences were identified, there are both visual and statistical indications that controlled burning, both with *Urban Suppression* and with *Urban and Wildland Suppression*, may reduce the mean and maximum number of burns in the model runs. These findings agree with literature that the implementation of controlled burning reduces burn intensity and fire risk through fuel management (Eales et al., 2016; Hong et al., 2023).

The impact of burned areas also influences the number of homes lost (Fig. 4d). The *No Management* strategy shows the highest number of burned homes and is statistically significantly different from all other strategies (Appendix B.2). Both *Urban Suppression* and *Urban Suppression with Controlled Burning* have numbers of burned homes so low that the values do not statistically differ from the extreme runs of *No Fire*. Overall, controlled burning in combination with Urban and Wildland Suppression gives the best outcomes for burned areas and homes lost according to our model.

**Fig. 6:**
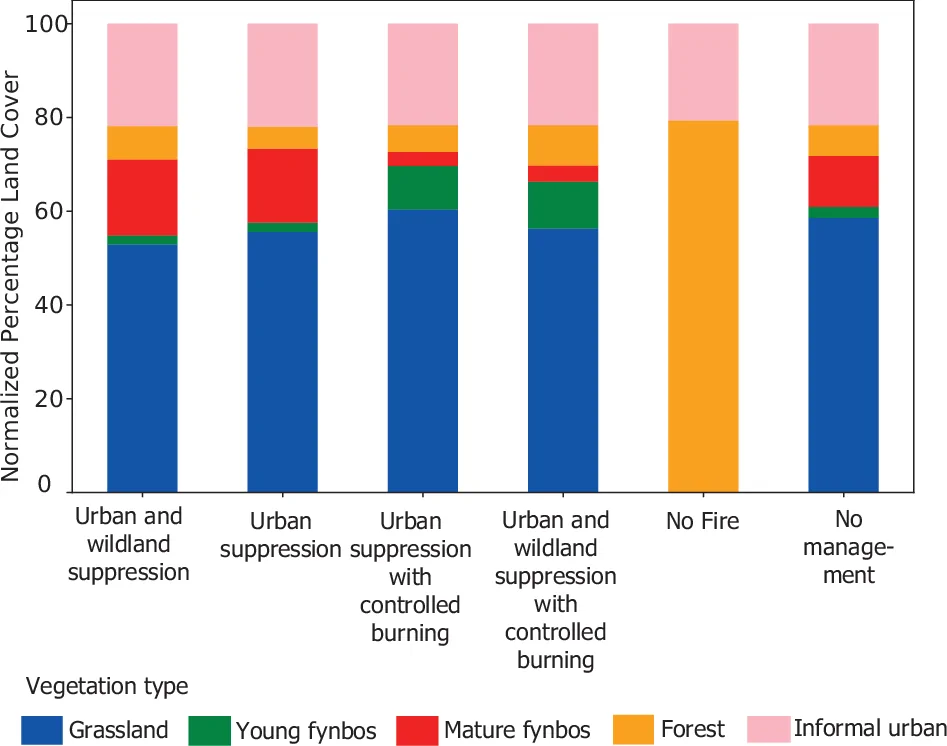
Percentage land cover outcomes per model settings (may not total to 100% as it is averaged percentages over multiple runs)

These results must be interpreted with caution. Our model output is based on the simple fire module that is employed. Generally speaking, the simulation outcomes do not represent real fires but rather more general stylised fire patterns. These patterns are an outcome of fuel load and fuel continuity. Furthermore, ignitions were modelled as a statistical process based on the fire occurrences and large fire events in the COCT fire incidence database (GeoTerraImage, 2016). Thus, ignitions are either extinguished right at the start or left to run their course. This is not a true representation of reality, where residents and firefighters would be actively fighting fires to slow or extinguish a fire. Seasonality is also not considered in our fire module, which may also influence the fire patterns and outcomes for fynbos. While this does mean our model may overestimate burned area, it was calibrated to match the fire patterns as indicated in the COCT fire database and the broader COCT fire patterns shown in Christ et al. (2022b) (also see Appendix A.1). If more detailed data is available, it would be possible to use Rothermel’s model (Andrews, 2018), FARSITE (Finney, 1998), or BEHAVE (Andrews, 1986) to use a more realistic fire model. Conceptually, more detailed data would be required, including local climate and vegetation data and a more detailed approach on how vegetation is modelled. Technically, using these other models within the framework would mean building a Rothermel implementation in NetLogo, or a novel way to pause NetLogo and run the FARSITE or BEHAVE model. In any case, a firefighting model would still have to be developed and parameterised. The added level of detail must match both the available data as well as the required detail to answer the question under study.

### Impact of Management Strategy on Vegetation Outcomes

In our simulations, all fire-inclusive strategies had fynbos present at the simulation end (Fig. 6). The largest area of young fynbos was observed in strategies with controlled burning, these strategies were statistically significantly different from all other strategies (Appendix B.1). The only exception here is the comparison between *Urban and Wildland Suppression* and *Urban and Wildland Suppression with Controlled Burning*. In contrast, strategies including controlled burning showed the smallest area of mature fynbos. This is expected as controlled burning aims at burning fynbos soon after maturity to reduce the overall fuel load while leaving viable fynbos seed. *Urban and Wildland Suppression* and *Urban Suppression* had the highest level of mature fynbos. Nearly all strategy comparisons with respect to mature fynbos were statistically significantly different.

In our model, the ability of fynbos to survive in a high suppression regime can be linked to the fire patterns, where fire escapes suppression and burns the fynbos while it is still viable. This resilience was not expected in our modelled environment but relates to the inevitability of fire in the case of an ignition in a fire-prone region with high fuel loads (Batcheler et al., 2024). However, this does not mean that all individual fynbos species would survive in such a situation. Our vegetation module aggregated all fynbos species and assumed a 7–20-year fire return period to represent fynbos viability. In reality, fynbos species differ in preferred return periods, from reseeders to resprouters, as well as the impact of climatic variability (Van Wilgen, 2013a, ; Bond, 1980). Our fire module does not account for fire seasonality and intensity, which would have an impact on species survival and is directly linked to fuel loads (Van Wilgen, 2013a).

All model runs except for the extreme *No Fire* run showed a considerable proportion of grassland and a share of forest similar to model initialisation. Thus, in our model, some forest areas are able to escape fires. Overall, our vegetation module was very simple, taking only basic categories for vegetation classes and simplistic succession processes. This was a deliberate choice to avoid building an unmanageable complex model and to keep data input requirements low. The aim was not to create the most realistic model, but to highlight how the entire system interacts and impacts on each other by bringing vegetation outcomes and urban outcomes into one modelling space. Future applications may focus on specific fynbos species and the local climatic variables that influence the maturation rate of these. Invasive species should also be included to show the impact fire has on their propagation. The period from fynbos senescence to forest also requires more detail, in our implementation, it is treated as a long-term state that fynbos cannot recover from. The inevitable spread of seed from one location to a neighbouring location when fire regimes improve was also not included in our model and will likely have a strong impact on how real fynbos succeeds after fires, especially in changing fire regimes.

### Impact of Management Strategies on Household Number and Type Distribution

**Fig. 7:**
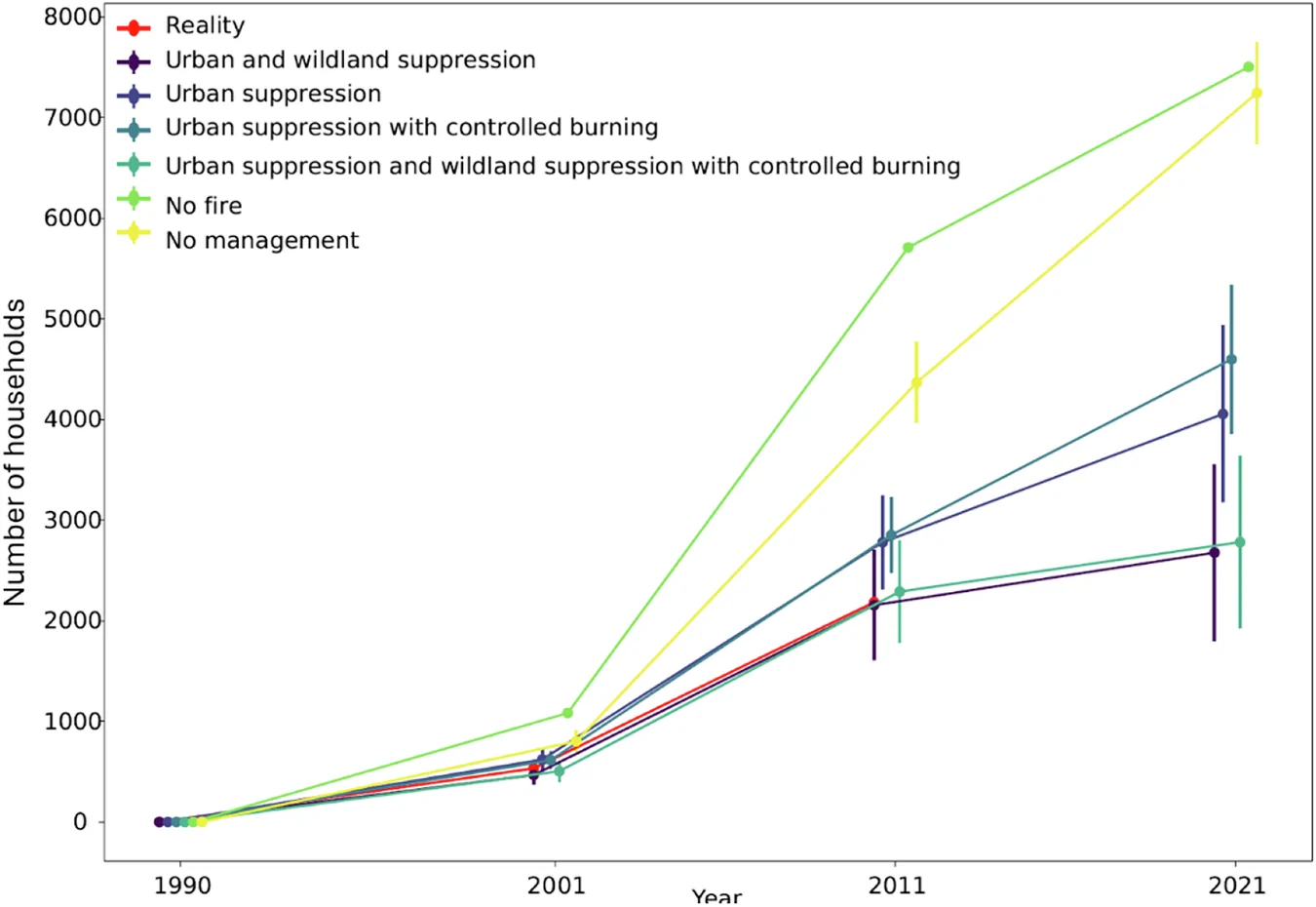
Number of households remaining in IY with error bars per management strategy in 1990, 2001, 2011 and 2021 (error bars denote variance between model runs with the same settings)

In our model, the various management strategies show counter-intuitive effects on total household numbers (Fig. 7) with most differences being statistically significant according to post hoc Tukey’s tests (Appendix B.3).

While *Urban and Wildland Suppression* and *Urban and Wildland Suppression with Controlled Burnin*g closely mirror census data, all other strategies show considerably higher total resident numbers in IY. Household numbers in *No Management* is only surpassed by removing fire from the system. These dynamics contrast with the expectation that with increasing fire activity, more residents would leave IY. This counter-intuitive model outcome is due to how residents’ behaviour was parameterised: Parameters influencing whether residents stay or leave the settlement are based on a comparison of a pre-and a post-fire survey (Christ et al., 2022a, 2023). The comparison of these surveys led to the—at first glance – surprising result that residents are less likely to leave immediately after a fire. These empirical findings are, however, in line with Jensen et al. (2014) and Lu et al. (2015), who found that repeated exposure to hazards and risk information can lead to a reduction in hazard perception. Other literature on fire perception found that residents’ actions do not always align with what is expected (Dupey and Smith, 2019). Residents’ decision-making aligns with expectations until a certain level of exposure, beyond which decision-making becomes less predictable and other processes such as experience, overestimation and underestimation of risk may play a role (Hertwig and Erev, 2009). Several studies on high flood risk areas also indicated there may be further reasons for residents to stay in flood-prone areas, such as social ties, perceived economic or social hardships in other locations or lack of information on the hazardous nature of the location or perceived safety after an event (Addo and Danso, 2017; Hemmati et al., 2021; Platt et al., 2020). As the likelihood of wildfires and floods is likely to increase in the future, it will be very important to better understand residents’ coping strategies and motivation for action.

**Fig. 8:**
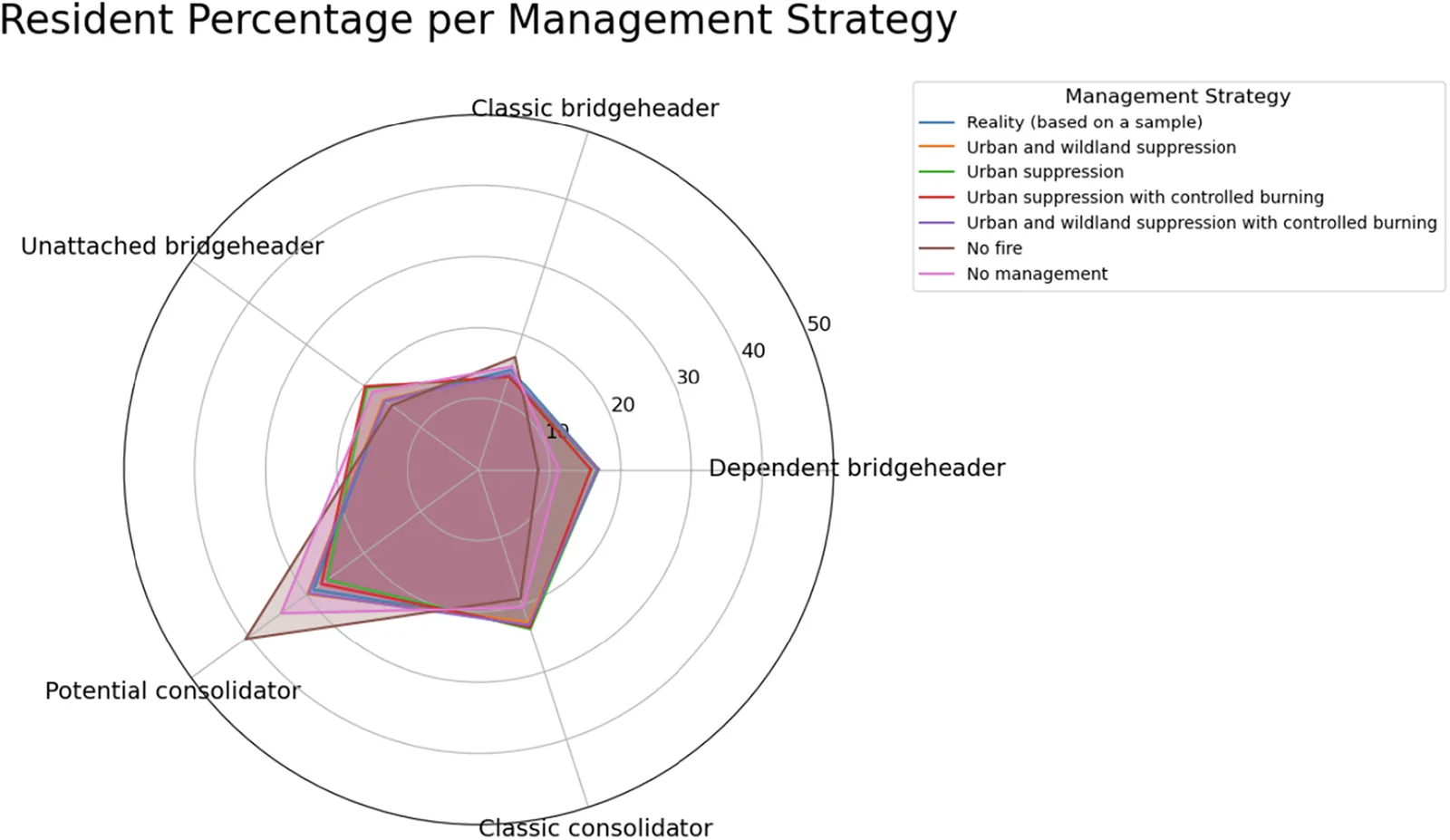
Spidergram for resident percentage per management strategy

Fire management strategies also influence the types of households remaining in IY in the model (Fig. 8). Dependent bridgeheaders are statistically significantly less frequent under *No Management* and in the case of *No Fire*, all other strategies show values close to the survey (Fig. 8, and Appendix B.3). In contrast, Classic bridgeheaders are more prevalent under these strategies. Unattached bridgeheaders are least prevalent in *No Fire*, and most common in *Urban Suppression with Controlled Burning* and *Urban Suppression*, with many statistically significant differences (Appendix B.3). Unattached bridgeheaders find IY attractive for reasons other than employment, and the lower hazard strategies may encourage them to stay. This could be indicative that more frequent fire may lead to classic consolidators taking more protective measures, whereas less frequent fires lead to greater losses due to longer accumulation of assets for those with more to lose, such as classic consolidators and unattached bridgeheaders. In Fig. 8, it is evident that potential consolidators are frequently the highest percentage of residents in IY, especially with lower risk; more of these would remain in IY, seeing it as an area to build and potentially become consolidators. The strength of attraction in terms of employment, however, keeps many residents in IY regardless of the fire hazard. This can be linked to the suggestion that informal settlements are a housing solution, despite potential hazards, because they provide benefits that potential alternatives may not (Turner, 1977).

While we used the decision-making theory as proposed and parametrised by Christ et al. (2023), the framework and the household model allow for other theories to be implemented. Users could simply redo the survey as done by Christ et al. (2023) to parametrise for a different study area, or completely replace the theory with another, such as rational choice theory (Moscati and Tubaro, 2011), bounded rationality (Yun et al., 2022), or theory of planned behaviour (Hagger et al., 2022). The theory should be tailored to the hazard under study, the study area and the outcomes of interest.

### Challenges in Implementing a Socio-environmental System Framework

As is common in ABMs integrating multiple scientific domains, the challenge lies in balancing generality, realism and precision. Our framework is intentionally generic and modular, allowing more complex modules in exchange for the relatively simple modules we employed. Even with these simple modules, modelling work ran into two recurring and related difficulties.

Firstly, many of the inputs required for modelling were not available in ready-made datasets. They had to be pre-processed from secondary datasets, often by using custom processing methods, as shown in the appendices and ODD + D documentation. Secondly, many human behaviour and ecological theories commonly accepted in practice are not readily usable in their qualitative form. They had to be translated into mathematical parameters with decision rules, often revealing ambiguities that had to be solved before the model could be implemented. These two categories of modelling challenges are also at the core of many decisions to use simpler modules rather than more complex modules that may bring in more data challenges and uncertain datasets. Finally, socio-ecological systems encompass feedback loops between ecological and social components. Our model incorporates a feedback loop from the experience of a fire to changing perceptions. Thus, a causal relationship between an experienced fire and changes in behaviour needed to be established. Despite the existing number of theories on human decision-making and the opportunity to conduct two surveys in the case study, this was hard to establish for at least two reasons. First, capturing human behaviour is essential, yet difficult to represent through a single theory or small ensemble of frameworks (Schlüter et al., 2017). We addressed this by combining PMT, place attachment, and Turner’s (1977) residential typology, as no single theory fully captured the complexity of the decision-making we observed. PMT and place attachment are not the only predictors of decision-making under extreme conditions; theories such as prospect theory and expected utility theory or opportunity-threat theory may also contribute; however, it was impractical to incorporate all theories into one model (Mishra, 2014; Pandey, 2018). The study also assumes that larger, uncontained fires are the primary concern, potentially overlooking the impact of smaller ignitions that were only statistically dealt with in our model on residents’ perceptions and behaviour. Research has also shown many residents believe their homes are unlikely to burn (Pharoah, 2009). Thus, the model may not fully capture the decision-making of all residents, particularly those only indirectly affected by fires.

Secondly, the survey design did not allow us to interview the same interviewees again, partly due to the location of the study and the impact of COVID-19; thus, changes in perceptions were only inferred from sample means. While our simulation results for urban residents under extreme conditions exhibit unexpected patterns, these align with literature on decision-making under risk, noting a difference between decision-making on rare events and frequent events (Hertwig and Erev, 2009). Also, another study found that an action needs to be seen as an effective resolution of the problem to be implemented (Dupey and Smith, 2019). We assumed that causalities of fire experience change perceptions based on the empirical data; however, longitudinal studies and theories about how perceptions change due to experiences of hazards are needed to better substantiate such feedback in socio-environmental systems.

Socio-ecological systems comprise several interlinked modules. In our case, we combined modules on residents’ decision-making, vegetation dynamics, fire ignitions and fire spread. For each module, we had to decide which level of detail to choose, whether to use an existing model and which simplifications to make. The trade-off here is between a more realistic representation, possibly closer to actual processes, and the additional effort of parameterising a more complex module. In most cases, we opted for simple solutions, with the idea of focussing on the interlinkages and the overall system behaviour instead. Therefore, the modules on vegetation dynamics, fire ignitions and fire spread have limitations as already described in detail above: There is no impact of fuel load on firefighting capabilities, vegetation is represented by ‘generic’ vegetation, without distinguishing between fynbos species or representing other, also invasive, species. For the fire model, we chose the Fisher et al. (2021) model as it was a NetLogo-based fuel-based fire model that required little vegetation parameters; while more complex models may be available, they would also have meant a more detailed vegetation model. We deliberately decided to include a somewhat more detailed representation of residents’ decision-making to pay attention to the vulnerabilities of the population in the area. Of course, others may well make other choices when conceptualising a socio-ecological system.

### Future Research

As an exploratory study integrating fire management, landcover change and individual households’ location choice into a modelling framework, we identified several areas for future research.

First, the resident module can be expanded to include different decision-making strategies, possibly varying them based on time since fire. Theories such as the theory of planned behaviour, expected utility theory, or prospect theory might be applied differently immediately after a fire versus during longer post-fire periods. Furthermore, extending the module to include formal urban residents and the factors influencing their decision to remain at a hazardous WUI could broaden the scope of the resident module. Ultimately, we recommend a longitudinal panel study to investigate how multiple events change residents’ perceptions and to follow up with residents that leave the settlement.

Further research should explore features directly relevant to residents and policymakers, such as incorporating monetary valuations of fire-related losses and management costs. Having active firefighters would improve these cost calculations, as firefighters are expected to be more effective with reduced fuel loads. Methods of maintaining urban fuel loads, such as wider roads and their implications for urban fires, should also be investigated. Furthermore, this type of research can also be used for planning the location of fire resources such as water reservoirs to have the most impact (Arabatzis et al., 2024).

The vegetation and fire module implementation are linked in complexity. Researchers wishing to use this framework must replace the fire module with a model appropriate for their study and should not simply reuse the one we have. Future work may also combine a more complex vegetation module with a more complex and realistic fire module. Future researchers here would have to choose their objective for outcomes and match the level of detail across these two modules to account for their parameters under study.

We invite further collaboration from fire scientists, urban planners and social scientists to refine the proposed framework. The Fire-WCISG Framework can evolve into a comparative tool for WUI systems worldwide, helping to test hypotheses, inform management trade-offs and connect empirical and modelling research in fire-prone and fire-dependent landscapes.

## Conclusions

The primary contribution of this work is the development of a modular agent-based framework for analysing coupled social-ecological fire management dynamics at the WUI. Fire-WCISG is a framework to model interlinkages of informal settlements at the WUI with vegetation and fires. To implement this framework, we chose mostly simple modules grounded in empirical data and literature. The results of this implementation need to be seen in the light of the limitations each individual module has. Still, they indicate room for further research, both empirically and in simulations.

Our framework implementation illustrates how interactions between fire, vegetation and residents may lead to different system-level outcomes. Results could indicate that fire-dependent species, such as fynbos, may exhibit better resilience to changing fire management regimes than expected. While this may partly be due to simplifications we made for the vegetation dynamics, it emphasises the importance of studying the impacts of fire management strategies on the whole system. Such studies ideally also encompass the impact of fire management not only on native (here: fynbos) species but also invasive species and the dynamics between native and invasive species.

Suppression-focused strategies without controlled burning may support some fire-dependent vegetation survival, but neglecting fuel accumulation can lead to large destructive fires causing significant damage to homes and risk to human life. In the model, the absence of any fire management still produces viable fynbos and maintains a low wildland fuel load but at the cost of increased home losses due to unmanaged fires. As this example highlights, the framework may facilitate a joint discussion of fire management strategies on outcomes across domains so that perspectives of different stakeholders may be heard.

Our modelled resident outcomes displayed great variability. Although resident behaviour was empirically parameterised, high fire frequencies led to counterintuitive results including increased population numbers and shifts in resident type distribution. This highlights the need for more research into understanding the processes that drive residential decision-making in vulnerable informal settlements. It also highlights the difficulty in moving from theory of decision making to modelled decision making.

Fire-WCISG: the model is released as open-source software, enabling researchers to expand it and incorporate alternative or multiple decision-making strategies, vegetation modules, and fire modules. This work demonstrates the ability and complexity of decisions of coupled models to investigate different phenomena based on existing knowledge.

## Supplementary information

Appendices

## Data Availability

The application with the required data to rerun the study is available at Comses (https://www.comses.net/codebase-release/33269c5b-6edb-41e4-8330-e0dd67ed85b0/).
