# Supplementary material for: Exploring Fire Management Strategies at the Wildland-Urban Interface with an Agent-Based Model Framework
Source: Environ Manage. 2026 Aug 27;76(9):288. doi: 10.1007/s00267-026-02561-7 (PMC13521984; doi:10.1007/s00267-026-02561-7)
Supplement: Supplementary file 1 [file 267_2026_2561_MOESM1_ESM.docx]

# Appendices

## Appendix A: Model calibration

### A.1 Calibration of fire suppression

To calibrate the fuel load we used the 1990-2011 burned area number of pixels of 20 runs averaged per model setting and compared them to the same period in the study area using the fire data from Christ et al. (2022b) as a reality baseline.

The suppression rations were varied around the reported fire suppression efficacy of 95% as communicated to the media by City of Cape Town officials (IOL NEWS, 2019). For each model set (20 model runs per settings) one of urban or wildfire suppression success percentage variables was varied between one of the following values: 95, 90, 85, 70. 60, 50. The overall patterns can be seen in Figure 14, multiple patterns were similar to the reality baseline.

Figure A.1 Burned pixels per wildfire suppression ratio

To narrow this down we also looked at the maximum burned pixels per model set and standard deviation per year, to find the settings that best match the reality dataset. Finally we plotted the sets on a normal distribution (Figure 17 and Figure 18), to evaluate which curve fits best. Table 16 highlights the best fits.

Figure A.2 Maximum burned area per model set

Figure A.3 Standard deviation of burned area between years per model set

Figure A.4 Normal distribution plots for burned area per model set part 1

Figure A.5 Normal distribution plots for burned area per model set part 2

Table A.1 Potential solutions also from the first graph

| Urban suppression | Wildland suppression | Comment |
| --- | --- | --- |
| 95 | 80 | A little below curve but still ok |
| 90 | 80 | Fits curve very well (best fits) |
| 90 | 70 | Fits curve very well (best fits) |
| 90 | 60 | Doesn’t fit well |

We chose 90 urban fire suppression and 80 wildland fire suppression as these were one of the two best matches across the tested statistics and closest to the self-reported figure of the City of Cape Town officials.

### A.2 Calibration of residents entering IY

Here, I miss a description of which parameter settings you varied and in what ranges and some results for the different settings.

This section describes the settings and results from various tests run to find the settings used in the Fire-WCISG model for Imizamo Yethu. The settings in the tests are seen below in Table A.2. Chosen is the settings that were chosen in the model. The number 1 and 2 represent the numbers for the first period and the second period respectively.

Table A.2 Settings for residents used in calibration. Chosen indicates the final settings used

| **Resident variable** | **Test 1** | **Test 2** | **Test 3** | **Test 4** | **Chosen** |
| --- | --- | --- | --- | --- | --- |
| Dependent bridgeheader 1 | 1 | 2 | 3 | 1 | 1 |
| Classic bridgeheader 1 | 1 | 2 | 8 | 5 | 4 |
| Unattached bridgeheader 1 | 1 | 2 | 5 | 3 | 3 |
| Potential consolidator 1 | 1 | 2 | 8 | 4 | 4 |
| Classic consolidator 1 | 1 | 2 | 8 | 3 | 4 |
| Dependent bridgeheader 2 | 1 | 5 | 10 | 12 | 7 |
| Classic bridgeheader 2 | 1 | 5 | 15 | 15 | 13 |
| Unattached bridgeheader 2 | 1 | 5 | 15 | 10 | 11 |
| Potential consolidator 2 | 1 | 5 | 20 | 40 | 37 |
| Classic consolidator 2 | 1 | 5 | 15 | 20 | 16 |

It can be seen that the chosen settings come the closest to the census period (Table A.3), these settings also come closest to representing the distribution Turner type residents found in Imizamo Yethu in 2021 by Christ et al. (2023)(Table A.4).

Table A.3 number of households in the model at selected census time frames

| **Years** | **Census** | **Test 1** | **Test 2** | **Test 3** | **Test 4** | **Chosen (difference)** |
| --- | --- | --- | --- | --- | --- | --- |
| Tick 0 (1990) | 0 (Settlement establishment) | 0 | 0 | 0 | 0 | 0(0) |
| Tick 44 (2001) | 532 | 235.1 | 329.2 | 960.3 | 470.6 | 554.9(+4.3%) |
| Tick 84 (2011) | 2183 | 316.5 | 743.7 | 2344 | 2594.3 | 2226.5(+2%) |
| Tick 124 (2021) | N/A | 383.4 | 987.25 | 2953.9 | 3879.2 | 3067(N/A) |

Table A.4 Turner type distribution in Imizamo Yethu 2021

| **Turner type** | **2021 survey %** | **Test 1** | **Test 2** | **Test 3** | **Test 4** | **Chosen (difference)** |
| --- | --- | --- | --- | --- | --- | --- |
| Dependent bridgeheader 1 | 18.5 | 35.5 | 20.5 | 25.2 | 22.9 | 16.2 (-2.3) |
| Classic bridgeheader 2 | 15 | 14.2 | 17.5 | 15.2 | 13.5 | 14.2(-0.8) |
| Unattached bridgeheader 3 | 16.1 | 20.5 | 24.4 | 21.6 | 12.9 | 17.0(+0.9) |
| Potential consolidator 4 | 27.6 | 10.0 | 13.3 | 15.2 | 27.5 | 29.7(+2.1) |
| Classic consolidator 5 | 22.8 | 19.9 | 24.4 | 22.9 | 23.5 | 22.9(+0.1) |

## Appendix B: Statistical significance testing of model output

## B.1 Vegetation results

The following tables represent Anova tests and Tukey's Honestly Significant Difference for comparing modelled results between modelled set.

Table B.1 Median Wildland Fuel Load Across Strategies

Table B.2 Number of patches with Continuous 4+ Year Fuel

Table B.3 Number of patches with Continuous 7+ Year Fuel Across Strategies

The following results relate to comparing the various vegetation types throughout the simulations and their Anova tests and Tukey's Honestly Significant Difference between modelled sets.

Table B.4 Percentage grassland

Table B.5 Percentage young fynbos

Table B.6 Percentage mature fynbos

Table B.7 Percentage forest

## B.2 Fire results

The following table relates to the Anova tests and Tukey's Honestly Significant Difference of the homes lost during fires in the different model runs.

Table B.8 Total homes lost

The below results relate to the number of times a pixel burned in the various modelled scenarios.

Table B.9 Statistics on average number of times a pixel burned per model run

| **Measured statistic** | **Anova p value** |
| --- | --- |
| Gini Coefficient | 0.286 |
| Mean | 0.153 |
| STD dev | 0.039 |
| Max | 0.048 |

The following results show the results for the Anova tests and Tukey's Honestly Significant Difference for the average different vegetation types at the end of the different simulation sets.

Table B.10 Statistical analysis for grassland

Table B.11 Statistical analysis for young fynbos

Table B.12 Statistical analysis for mature fynbos

Table B.13 Statistical analysis for forest

## B.3 Resident results

Below the results relate to the number of households present at various points in the simulation runs, between the strategies.

Table B.14 Anova for total households between management strategies at key steps (Year)

| **Step (Year)** | **P value** |
| --- | --- |
| 44 (2001) | p<0.001 |
| 84 (2011) | p<0.001 |
| 124 (2021) | p<0.001 |

Table B.15 Average percentage of household types per model settings in 2021 -Total may not add to 100% due to rounding (raw number of residents in brackets)

| **Household type** | **Reality (based on a sample)** | **Urban and wildland suppression** | **Only urban suppression** | **Only urban suppression with controlled burning** | **Urban and wildland suppression with controlled burning** | **No fire** | **No management** |
| --- | --- | --- | --- | --- | --- | --- | --- |
| **Dependent bridgeheader** | **16.9** | 16.8  (449.2) | 16.7 (662.2) | 15.8  (714.5) | 16.7  (464.7) | 8.4 (632.2) | 11.3  (817.6) |
| **Classic bridgeheader** | **14.8** | 14.2  (379.0) | 13.8 (562.5) | 13.8  (635.4) | 14.2  (397.1) | 16.7 (1255.2) | 15.3 (1109.4) |
| **Unattached bridgeheader** | **16.4** | 16.6  (451.3) | 19.5 (792.7) | 19.8 (909.6) | 16.4  (461.1) | 15.2 (1136.9) | 18.5 (1338.9) |
| **Potential consolidator** | **28.8** | 29.8  (786.8) | 26.4 (1084.0) | 27.4 (1274.0) | 29.6  (814.7) | 40.6 (3044.4) | 34.4 (2500.9) |
| **Classic consolidator** | **23.0** | 22.6  (609.9) | 23.6 (952.4) | 23.3 (1064.1) | 23.0  (642.9) | 19.1 (1433.5) | 20.4 (1478.8) |
| **Total** | N/A | 100  (2676.1) | 100 (4053.7) | 100.1 (4597.5) | 99.9  (2780.4) | 100 (7502.0) | 99.9  (7245.4) |

The below tables give the Statistical tests for the various scenarios and number of residents and Turner types presents in the model.

Table B.16 Anova results between Turner types based on strategies at the end of simulation

| **Household type** | **P value** |
| --- | --- |
| **Dependent bridgeheader** | p<0.001 |
| **Classic bridgeheader** | p<0.001 |
| **Unattached bridgeheader** | p<0.001 |
| **Potential consolidator** | p<0.001 |
| **Classic consolidator** | p<0.001 |

Table B.17 Tukey's Honestly Significant Difference for the number of residents in 2001

Table B.18 Tukey's Honestly Significant Difference for the number of residents in 2011

Table B.19 Tukey's Honestly Significant Difference for the number of residents in 2021

Table B.20 Tukey's Honestly Significant Difference for Dependent bridgeheaders

Table B.21 Tukey's Honestly Significant Difference for Classic bridgeheaders

Table B.22 Tukey's Honestly Significant Difference for Unattached bridgeheader

Table B.23 Tukey's Honestly Significant Difference for Potential consolidator

Table B.24 Tukey's Honestly Significant Difference for Classic consolidator
